# Microhabitat Segregation of Co‐Existing Nightjar Species in The Gambia

**DOI:** 10.1002/ece3.71656

**Published:** 2025-06-23

**Authors:** José‐María García‐Carrasco, Clive Richard Barlow, Carlos Camacho

**Affiliations:** ^1^ Department of Entomology Washington State University Pullman WA USA; ^2^ Department of Animal Biology, Faculty of Science University of Malaga Malaga Spain; ^3^ Birds of The Gambia Brufut Gardens Brusubi Western Coastal Region The Gambia; ^4^ Department of Ecology and Evolution Estación Biológica de Doñana (CSIC) Seville Spain

**Keywords:** ecological interactions, interspecific competition, niche partitioning, paddy field, West Africa

## Abstract

The coexistence of nocturnal bird species in tropical ecosystems remains poorly understood, primarily due to the difficulty of detecting and monitoring these elusive organisms. We studied the spatial distribution of two ecologically similar nightjar species, the Long‐tailed Nightjar *Caprimulgus climacurus* and Standard‐winged Nightjar *Caprimulgus longipennis* , in The Gambia. Under the assumption that competition is particularly intense among closely related species, we aim to investigate the degree of spatial overlapping between the two nightjar species as a preliminary test of spatial niche partitioning in nightjars. During the early dry season of 2021, we recorded the location of nightjars sitting on dirt tracks at night inside and outside of a rice field area in the Central River Region in The Gambia. We analysed the abundance, density and spatial distribution pattern of nightjars to determine if the two species segregate in space along tracks or occur together. The density of Long‐tailed Nightjar was higher than that of Standard‐winged Nightjar (9:1), and density was higher inside the rice field (4.41 nightjars/km) than outside of it (0.58 nightjars/km). The spatial analysis suggested that both species tended to co‐occur at the landscape scale (> 500 m) and avoided each other over finer spatial scales (< 500 m). This study identifies, for the first time, the potential for niche partitioning and spatial segregation at the community level in caprimulgids, although larger scale studies are needed to confirm the generality of the observed patterns. Moreover, our research highlights the magnitude of the utilisation of rice fields for foraging by nightjars in this novel study of this habitat type in The Gambia. Understanding the role of land use management patterns and the interaction of poorly studied nocturnal birds in (sub)tropical areas has important implications to support decision‐making in species conservation planning and land management.

## Graphical abstract

We studied the spatial distribution and habitat use of Long‐tailed Nightjar ( *Caprimulgus climacurus* ) and Standard‐winged Nightjar ( *Caprimulgus longipennis* ) in The Gambia to test the hypothesis of spatial niche partitioning. Our findings reveal that while the species co‐occur at the landscape scale, they avoid each other at finer spatial scales, highlighting potential niche partitioning. This study underscores the importance of rice fields as key foraging habitats for nightjars and provides insights into the implications of land‐use management for nocturnal bird conservation in tropical ecosystems.

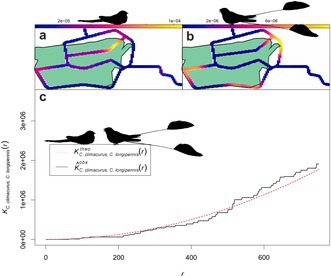

## 1 Introduction

Classical niche partitioning theory states that coexisting species partition limiting resources in spatial, temporal or trophic dimensions as a mechanism to reduce competition (Hutchinson 1959). Competition is particularly intense among closely related, morphologically similar species that use similar resources in a similar manner (Fox 1987). To lessen competition for resources and thus facilitate coexistence, species sharing common traits may forage at different times of the day or at different sites or target different prey species. Dietary, spatial and temporal niche partitioning is a long‐standing theme in ornithological field studies. These studies are focused mostly on communities of colonial seabirds (Procellariidae) and combine satellite tracking and isotopic information to characterise diet and spatial and temporal foraging patterns (Croxall and Prince 1980; Jessopp et al. 2020; Masello et al. 2010; Navarro et al. 2013, 2015; Petalas et al. 2024; Phalan et al. 2007). Classic examples of resource partitioning and its importance for community stability can also be found among non‐colonial species belonging to families as diverse as Meliphagidae (Ford and Paton 1976), Picidae (Alatalo 1978) or Emberizidae (Pulliam 1985). Nevertheless, studies of resource partitioning in nocturnal bird species are still scarce (but see Hayward and Garton 1988; Mehta et al. 2018), which thus limits our understanding of the structure of communities of nocturnally active birds (Gaston 2019; Park 1940).

Nightjars (Caprimulgidae) are crepuscular and nocturnal aerial insectivores that use the night sky as a background to detect the silhouette of prey (mainly aerial insects) and pursue them during short flights made most frequently from the ground (Bayne and Brigham 1995; Cleere 1998; Jackson 2002). Such a foraging strategy requires some sort of illumination source, and therefore, the temporal niche of nightjars is restricted to periods of semi‐darkness, especially in the form of moonlight (Jetz et al. 2003; Mills 1986, 2008). Constraints on foraging due to limitations of visual orientation in dark conditions could restrict possibilities for expansion of the dietary and temporal niche of nightjar species and result in similar foraging patterns across the group concerning these dimensions (Bayne and Brigham 1995; Evens et al. 2020; Jetz et al. 2003; Mills 1986). Thus, interspecific partitioning of foraging space should be a critical dimension of niche differentiation in nightjar communities, but no field data are previously available to address this question.

This study investigates the spatial patterns of nocturnal foraging of ecologically similar nightjar species that coexist in an irrigated rice scheme in The Gambia. Both Long‐tailed Nightjar ( *Caprimulgus climacurus* ) and Standard‐winged Nightjar ( *C. longipennis* ) are confirmed breeders in the study region (Barlow et al. 1997). However, direct observations of eggs and pulli are rare for both species in The Gambia. Both species perform regional movements, but detailed information about their nature and magnitude is also lacking. The diet of the Long‐tailed Nightjar and Standard‐winged Nightjar remains little studied, but anecdotal evidence from stomach content analyses and field observations suggests that they exploit the same food resource and use the same foraging mode and method of prey capture (Cleere and Kirwan 2020; Cleere 2020; Jetz et al. 2003). They both feed primarily on moths (Lepidoptera) taken in flight, since this is the predominant insect order during the nighttime (see figure 2 in Jetz et al. (2003)), although they may also occasionally feed on the ground on terrestrial insects, mainly Orthoptera (authors' pers. obs.) and Coleoptera (see Jackson 2002 and references therein). Both species almost completely overlap in time of foraging activity, as demonstrated by a study investigating nocturnal, lunar and seasonal allocation of activities in a small open area of the Guinea savannah zone (Jetz et al. 2003). Both the Long‐tailed and the Standard‐winged Nightjar become active shortly after sunset, there is increased nocturnal foraging activity during full moon periods to leverage lunar illumination, and reproduction is timed to match a seasonal surge of prey availability soon after the onset of the wet season (Jetz et al. 2003). They both use trails, tracks and roads as foraging and resting sites during the night, but a detailed analysis of microhabitat use patterns in nightjar communities is lacking despite the potential importance of segregation in microhabitat use as a mechanism to facilitate the co‐existence of competing species. Based on occurrence and abundance data collected during nocturnal transect counts, we analyse the fine‐scale spatial distribution of Long‐tailed Nightjar and Standard‐winged Nightjar along dirt tracks criss‐crossing the study area and discuss the results in the context of spatial segregation and niche partitioning. Although limited in temporal and spatial scope, our study aims to offer valuable baseline insights that may guide future investigations into nightjar coexistence.

## 2 Methods

### 2.1 Study Area

**FIGURE 1:**
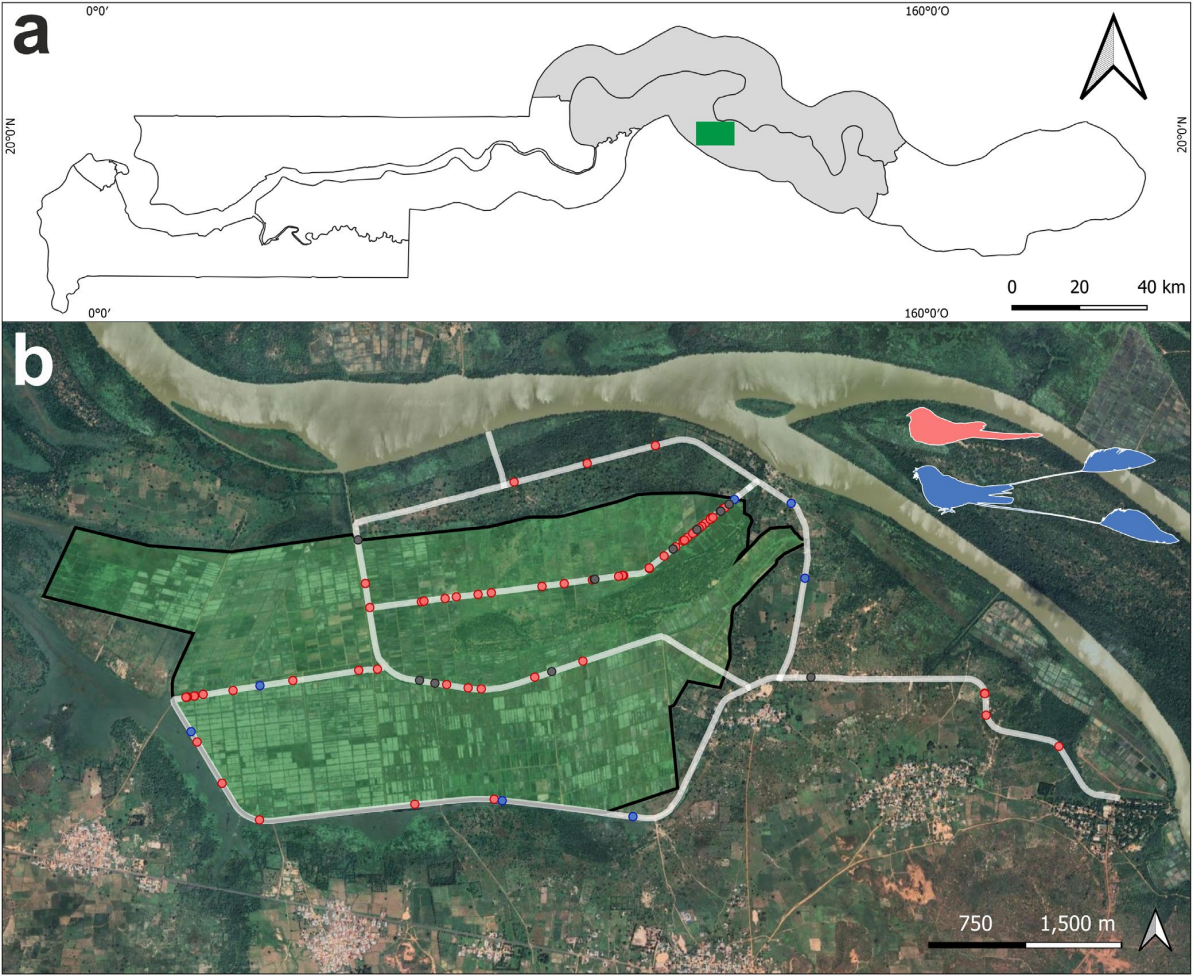
Map of the study area. (a) Location of the Pacharr rice fields (green polygon) in Central River Region (grey area) of The Gambia. (b) Pacharr rice fields (greenish polygon) and the surveyed transects (white lines). The transects along the edge are still within the rice field boundaries. Red dots indicate records of Long‐tailed Nightjar ( *Caprimulgus climacurus* ), blue dots indicate records of Standard‐winged Nightjar ( *Caprimulgus longipennis* ), and black dots represent records of unidentified nightjars.

The study was conducted on the extensive Jahally‐Pacharr irrigated rice scheme (14.9518° N, 13.5598° W) established in the mid‐1950s (and modernised in the 1980s): situated in the Central River Region in The Gambia with an area of approximately 1000 has (Figure 1). The scheme borders the Gambia River on the south bank, which flows permanent fresh water in this area (Minteh et al. 2019). During the study period, most of the fields were flooded.

### 2.2 Sampling

Many nightjar species are attracted to roads and trails at night (Jackson 2002), so road and trail transects are one of the most commonly used methods for nightjar monitoring (Camacho 2013; Camacho et al. 2017; De Felipe et al. 2019; Pomeroy et al. 2013). Using a bicycle for transport and with the aid of a handheld 1000 lumen torchlight, a GPS equipment device, and 8 × 42 binoculars, standardised nocturnal transect counts of trail and road‐sitting nightjars were conducted to establish species identification and collect location data. Nightjars foraging and resting on trails and dirt tracks were found by scanning the ground using the 1000 lm torchlight during six nights on 7–13 December 2021 (14%–73% moon‐face illuminated; daily minimum temperature: 19°C, maximum temperature: 38°C). Time‐concentrated sampling (i.e., a series of counts on consecutive days) precludes generalisation to other times and places but is considered a cost‐effective approach for assessing fine‐scale space use patterns of nightjars using roads to rest and forage since seasonal changes in environmental factors, such as food availability, predator abundance and ambient temperature, can influence nightjar use of roads and thus confound occurrence data obtained from direct counts of road‐sitting individuals (Camacho et al. 2017; De Felipe et al. 2019). Surveys were conducted between 23 h 00 and 01 h 00. December is considered the early dry season with rainfall occurring mid‐June–mid‐October in most years (Barlow et al. 1997). The transects comprised narrow foot trails created by farmers inside dry harvested rice fields and along a network of adjoining wider dirt tracks used by motorised vehicles and donkey carts which crisscross the entire rice field project. The distance travelled in and out of the rice fields was recorded to calculate the nightjar density per kilometre.

Sightings were identified to species level on 85% of all encounters, and a GPS reading was taken and stored. In some cases, nightjars flew off before species‐level identification was possible, so these observations were recorded as *Caprimulgus* sp. Nightjars often take a short flight as the observer approaches and land further along the transect. To avoid counting the same individual more than once and thus minimise overestimation of nightjar abundance, when a nightjar took flight on approach, the next 100 m were not considered for the subsequent nightjar found.

### 2.3 Data Analysis

We calculated the Kilometric Abundance Index (KAI), a metric that facilitates comparing the abundance of a species across different locations or different species within the same location (Preatoni et al. 2012; Tellería 1986; Vincent et al. 1991). The geo‐positioned bird records were cartographically represented using the QGIS (http://www.qgis.org) program as well as the geo‐position of the surveyed tracks. We used the R package SPATSTAT (Baddeley and Turner 2005) to analyse the spatial point pattern distribution of both species across the study area. Since both inside and outside the rice field plots, sampled areas consisted of tracks, we created a 5‐m buffer (or influence area) to represent the width of these traversed tracks. The resulting polygon, formed by several lines (from the different tracks) and with a width of 5 m, was used as the analysis window (white lines in Figure 1b, and *a* in the function below), from which the following spatial analyses were conducted. We performed a Kernel Smoothed Intensity of Point Pattern analysis to assess the spatial distribution and density of nightjar occurrences across the study area (Baddeley and Turner 2005). This method allowed us to visualise the concentration of points and identify areas of high and low occurrence of each species, providing insights into the spatial patterns of bird distributions and potential hotspots of activity. Moreover, we performed a cross *K*‐function analysis to determine whether the fine‐scale distribution of the two species was aggregated or, on the contrary, segregated in space. The cross *K*‐function is a generalisation of the Ripley's function to multiple point pattern, which reads as follows:

$$\hat{K}\left(r\right)=\frac{a}{n\left(n-1\right)}\;\sum_{i}\sum_{j}\;I\left(d_{\mathrm{ij}}\leq r\right)e_{\mathrm{ij},}$$

where *a* represents the area of the window, *n* is the total number of data points; *d*_*ij*_ is the distance between the two points, and *I* (*d*_*ij*_ *≤ r*) is the indicator that equals 1 if the distance is less than or equal to *r*; the term *e*_*ij*_ is the edge correction weight, automatically computed using the default isotropic edge correction method provided by the package; and *r* is the search radius or distance (Baddeley et al. 2016; Ripley 1976). The reference line used for comparison corresponds to the theoretical expectation of the *K*‐function under complete spatial randomness, which is given by *K* (*r*) = *πr* ^2^ in two‐dimensional space. If the line of the cross *K*‐function is above the reference line that denotes a random distribution (homogeneous Poisson), it would indicate spatial clustering and co‐occurrence of species (*K̂*_*C.longipennis*,*C.climacurus*_(*r*) > *K*_Poisson_(*r*)). Conversely, if the line of the cross *K*‐function is below the reference line (*K̂*_*C.longipennis*,*C.climacurus*_(*r*) < *K*_Poisson_(*r*)), then individuals of the Long‐tailed Nightjar and the Standard‐winged Nightjar may be assumed not to be present in areas where the other species is found. To reduce detection bias resulting from greater visibility along the sampling tracks compared to the surrounding crop areas, and better reflect spatial segregation patterns, analyses were restricted to direct observations recorded along the sampling tracks. The short duration of the study, together with the small sample size obtained from it prevents the use of formal statistical testing of clustering or segregation patterns, so descriptive statistics are presented.

## 3 Results

During the six nights of sampling, a total of 35.21 km were surveyed, involving 18.90 km outside the rice fields and 16.31 km inside the rice fields. A total of 96 nightjar observations were made throughout the entire sampling period. Of these, 83 were included in our analysis; 13 sightings were excluded because of the possibility of repeat counting when individuals flushed and landed further along a transect. This resulted in an overall density of 2.36 nightjars/km. We recorded 64 Long‐tailed Nightjars (1.82 birds/km) and 7 Standard‐winged Nightjars (0.20 birds/km). On seven occasions, Long‐tailed Nightjars were found resting together in twos within a radius of 2 m, and in one case, three birds were found resting together. All Standard‐winged Nightjar sightings occurred as singletons.

**FIGURE 2:**
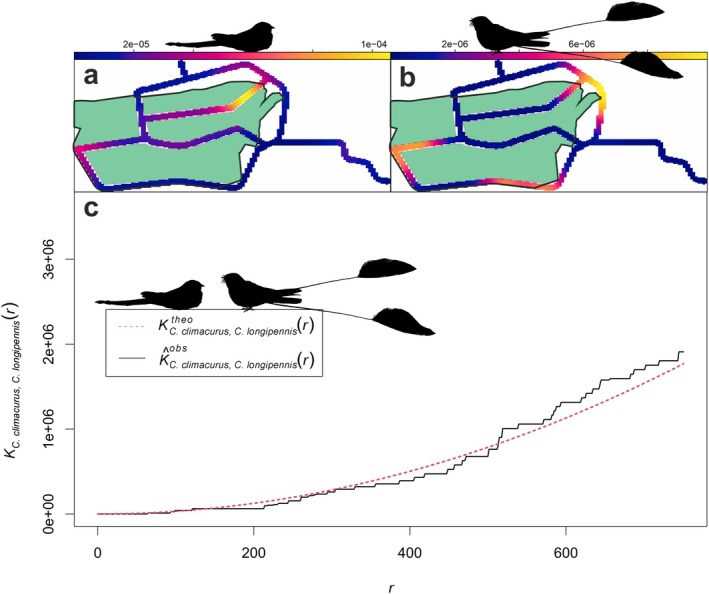
Spatial analysis of Long‐tailed Nightjar ( *C. climacurus* ) and Standard‐winged Nightjar ( *C. longipennis* ) across all surveyed tracks and over the six nights. (a) Point density of Long‐tailed Nightjar; (b) point density of Standard‐winged Nightjar. The green polygon represents the extension of the rice field. (c) Results of the cross *K*‐function analysis. The red line represents the distribution that points would follow if they were randomly distributed. The black line is the *K*‐cross function line.

Nightjars were over six times more abundant on tracks inside the rice fields than on the adjacent dirt tracks outside of them: 72 (4.41 birds/km) versus 11 individuals (0.58 birds/km), respectively. In the case of Long‐tailed Nightjar: 57 (3.46 birds/km) were inside and seven (0.37 birds/km) outside. The Kernel Smoothed Intensity of Point Pattern analysis provided a more in‐depth picture of the Long‐tailed Nightjar's space use, showing that it primarily utilised the area within the rice field (Figure 2a). In the case of Standard‐winged Nightjar, 4 (0.25 birds/km) were inside and 3 (0.16 birds/km) outside. Notably, three of the four individuals inside the rice field were located in the edge of the field (Figure 1b), making a peripheral use of the rice field (Figure 2b). Based on the position of the cross *K*‐function line relative to the reference line (random distribution), there appears to be some degree of spatial segregation at short distance scales (< 500 m) and spatial clustering at longer distances (> 500 m) (Figure 2c).

## 4 Discussion

Niche partitioning has been documented in many space‐use studies of diurnal birds (Alatalo 1978; Hart et al. 2021; Jessopp et al. 2020; Kent and Sherry 2020; Traba et al. 2015), but the phenomenon remains understudied for nocturnal birds. In this study, we surveyed two congeneric nightjars in The Gambia to examine differences in their use of space during foraging as a first step to understand potential ecological interactions in these species. Despite the short duration of the study and the small sample size for one of the species, our study suggests a potential trend of co‐occurrence of Long‐tailed Nightjars and Standard‐winged Nightjars at a larger scale and spatial segregation in the use of foraging microhabitats at a finer scale.

The spatial patterns observed in our study provide preliminary insights into the potential partitioning of foraging space of Long‐tailed Nightjars and Standard‐winged Nightjars at the study site. Our findings suggest that these species use distinct microhabitats for foraging, with Long‐tailed Nightjars predominantly occupying the interior of rice fields, whereas Standard‐winged Nightjars are more frequently observed along the edges. This spatial segregation may reflect regional coexistence and small‐scale partition of foraging space, although the observed pattern could also be influenced by substantial differences in the local densities of the two species. The microhabitat segregation pattern of nightjars suggests that interspecific competition for space may occur in the study area, but caution is required in interpreting co‐occurrence data as an indication of non‐trophic interactions, such as competition for space or time (Blanchet et al. 2020). For instance, the presence of a nightjar species could influence the occurrence of the other, resulting in a spatial segregation of the species, but a similar spatial pattern might emerge if different species prefer different microhabitats for foraging. These findings suggest that the observed fine‐scale segregation may serve as a mechanism facilitating the shared use of regional resources (e.g., foraging space), but behavioural observations are needed for a mechanistic interpretation of the observed spatial pattern.

It is important to recognise that the short duration of the survey, the limited spatial extent of our study area, and the natural scarcity of one of the study species in this area reduced the sample size available for study and precluded formal testing of statistical significance. Multiple counting of some individual nightjars also cannot be ruled out, although this is unlikely to be common in densely populated areas due to the typically high turnover of individuals along roads (Camacho 2013). Furthermore, the brief observation period may coincide with specific seasonal or weather‐related conditions, potentially influencing detectability and activity patterns. Some records that could not be identified at the species level added uncertainty to the results. Future studies, incorporating replicated data from multiple sites, extended sampling periods and analyses of prey availability and preference are needed to confirm the validity and broader applicability of these preliminary observations and to further elucidate the ecological processes underlying niche partitioning in nocturnal avian species.

The transect counts conducted for this study provide novel information about nightjar use of rice fields and nightjar densities along soft linear developments, such as trails and dirt tracks, in Africa. During the six‐night period in our survey area, Long‐tailed Nightjars were more numerous than Standard‐winged Nightjars. This finding contrasts with another brief study in a savannah‐grassland plot of West Africa, in Comoé National Park, Côte d'Ivoire, during the same period in December and involving the same two species. Jetz et al. (2003) estimated a density of 0.31 bird/km for Long‐tailed Nightjar and 0.12 bird/km for Standard‐winged Nightjar, compared to our 1.82 and 0.20 birds/km, respectively. The differences were of an even greater magnitude if just the densities inside the rice fields are considered, that is, 3.49 and 0.25 bird/km, respectively. In Uganda, Pomeroy et al. (2013) conducted 1–3 annual surveys over 9 years (2004–2012) using a car around an airfield to count Standard‐winged Nightjar (January) and Pennant‐winged Nightjar (July) (Pomeroy et al. 2013). In that study, Standard‐winged Nightjar density was higher than in Pacharr rice fields and varied from 0.69 to 2.39 bird/km over the 9‐year study period. Since the time of year is similar in all the studies (December–January) and the distribution of both species studied covers Senegambia to Uganda (Cleere and Kirwan 2020; Cleere 2020), density differences may be due to migratory behaviour or environmental favourability for the different areas.

Despite being anthropic spaces, rice fields are becoming increasingly recognised as important habitats for both African and migratory birds (Wymenga and Zwarts 2010). Insects are noted, by West African farmers, to be causing severe losses of productivity in lowland rice fields (Adesina et al. 1994; Garcia et al. 2021). Therefore, farmers can benefit from insectivorous birds in their rice fields that provide control services for invertebrate crop pests (Garcia et al. 2021). Our work provides new foraging related information for two nightjar species in The Gambia. Comparable studies in rice fields across all seasons are suggested in all river regions across The Gambia and extended to other rice producing countries in West Africa. The agricultural landscape of rice fields and their borders would appear to afford favourable habitat for communal foraging by nightjars and this merits a fuller investigation. This would complement what is already known for the importance of West African rice projects for water birds (Wymenga and Zwarts 2010) and thus support decision‐making in conservation planning and management (Bartolommei et al. 2013).

In conclusion, our findings underscore the critical importance of investigating ecological niche components for declining species, such as in the case of many nightjar species across all continents. Multi‐species studies of nightjars are almost non‐existent. Such studies are essential to assess the differential vulnerabilities of species to interspecific competition and anthropogenic pressures such as extensive land‐use change and habitat fragmentation. Understanding these interactions and the ecological conditions influencing species' distributions at multiple spatial scales is needed to generate short‐term forecasts and project population dynamics under global change scenarios in order to inform conservation strategies.

## Author Contributions

**José‐María García‐Carrasco:** conceptualization (lead), data curation (equal), formal analysis (equal), investigation (equal), methodology (lead), visualization (equal), writing – original draft (lead), writing – review and editing (equal). **Clive Richard Barlow:** investigation (equal), supervision (equal), writing – review and editing (equal). **Carlos Camacho:** conceptualization (supporting), investigation (equal), methodology (supporting), supervision (lead), writing – original draft (equal), writing – review and editing (lead).

## Conflicts of Interest

The authors declare no conflicts of interest.

## Data Availability

All relevant data for this study are available within the article, including spatial data and census results.
